# Local Vancomycin Application Reduces Periprosthetic Joint Infections in Oncologic Megaprosthetic Reconstruction: A Retrospective Cohort Study

**DOI:** 10.3390/antibiotics14090952

**Published:** 2025-09-19

**Authors:** Andreas G. Tsantes, Dimitrios V. Papadopoulos, Stavros Goumenos, Petros Ioannou, Nikolaos Stavropoulos, Eleni Petrou, Ioannis G. Trikoupis, Christos Koutserimpas, Alexandra Mpakosi, Vasileios A. Kontogeorgakos, Stefanos Bonovas, Panayiotis J. Papagelopoulos, Athanasios Tsakris, Argirios E. Tsantes

**Affiliations:** 1 Laboratory of Haematology and Blood Bank Unit, “Attiko” Hospital, School of Medicine, National and Kapodistrian University of Athens, 12462 Athens, Greece; epetrou@med.uoa.gr (E.P.); atsantes@med.uoa.gr (A.E.T.); 2 Microbiology Department, “Saint Savvas” Oncology Hospital, 11522 Athens, Greece; 3 Second Department of Orthopaedics, School of Medicine, National and Kapodistrian University of Athens, 11527 Athens, Greece; papadopo@med.uoa.gr (D.V.P.); nikstav@med.uoa.gr (N.S.); 4 First Department of Orthopaedics, School of Medicine, National and Kapodistrian University of Athens, 11527 Athens, Greece; stavros.goumenos@charite.de (S.G.); itrikoupis@med.uoa.gr (I.G.T.); vkonto@med.uoa.gr (V.A.K.); pjportho@med.uoa.gr (P.J.P.); 5 Department of Internal Medicine & Infectious Diseases, University General Hospital of Heraklion, 70013 Crete, Greece; 6 School of Rehabilitation Health Sciences, University of Patras, 26504 Patras, Greece; koutserimpas@upatras.gr; 7 Department of Microbiology, General Hospital of Nikaia “Agios Panteleimon”, 18454 Piraeus, Greece; ampakosi@auth.gr; 8 Department of Biomedical Sciences, Humanitas University, Pieve Emanuele, 20072 Milan, Italy; stefanos.bonovas@hunimed.eu; 9 IRCCS Humanitas Research Hospital, Rozzano, 20089 Milan, Italy; 10 Department of Microbiology, Medical School, National and Kapodistrian University of Athens, 11527 Athens, Greece; atsakris@med.uoa.gr

**Keywords:** periprosthetic joint infections, bone tumors, megaprosthetic reconstruction, vancomycin, local administration

## Abstract

**Background/Objectives:** Periprosthetic joint infections (PJIs) represent a serious complication following musculoskeletal tumor resection and megaprosthetic reconstruction. Local antibiotic administration may reduce infection risk by achieving high local drug concentrations. The aim of this study was to evaluate whether local vancomycin powder reduces postoperative periprosthetic infections in bone tumor surgeries involving megaprostheses. **Methods:** This retrospective cohort study included 276 patients who underwent bone tumor resection and megaprosthetic reconstruction. Study subjects were divided into two groups: the control group (n = 142) that received standard perioperative intravenous antibiotics, and the vancomycin group (n = 134) that received an additional 1 g of vancomycin powder locally at wound closure. Periprosthetic joint infections were defined using the 2018 International Consensus Meeting (ICM) criteria and monitored for 2 years. A multivariable competing risks regression model was used to assess the independent effect of local vancomycin on infection risk. **Results:** Periprosthetic joint infections occurred in 28 patients in the control group (19.7%) vs. eight patients in the vancomycin group (5.9%, *p* = 0.001). The most frequently isolated pathogens were coagulase-negative staphylococci (52.7%), followed by *Staphylococcus aureus* (22.2%). Among infected patients in the vancomycin group, only two had Gram-positive infections, suggesting efficacy against staphylococcal PJIs. The multivariable regression confirmed a significantly lower risk of infection in the vancomycin group (hazard ratio [HR]: 0.40, 95% confidence interval [CI]: 0.16–0.95, *p* = 0.040), while pelvic tumors were associated with a higher infection risk (HR: 5.82, *p* < 0.001). **Conclusions:** Our results indicate that local vancomycin may reduce periprosthetic infection rates in oncologic megaprosthetic reconstruction without added complications. Randomized studies are warranted to confirm these findings and refine dosing strategies.

## 1. Introduction

Recent advances in chemotherapy, modern surgical techniques, and improvements in the design of oncologic prostheses have significantly increased the number of limb salvage procedures performed for resection of bone tumors. Postoperative infection however remains one of the most worrisome complications of these surgeries, with a reported rate in the literature ranging from 2.2% to 50% [1,2]. This rate is significantly higher compared to the 0.5–2% infection rate observed in conventional total hip and knee arthroplasties [3,4]. The higher infection rate in oncologic surgeries may be attributed to their complexity, the prolonged duration, the extensive blood loss, the use of megaprostheses and allografts, and to the immunosuppression status of patients receiving cytotoxic chemotherapeutic agents [5]. Moreover, the megaprosthetic implants that are used in orthopedic oncology have a much larger metallic surface compared to the conventional implants that are used in hip and knee arthroplasties, providing a larger area for bacterial attachment and subsequent biofilm formation. The management of periprosthetic joint infections (PJIs) often requires multiple surgeries and prolonged antibiotic therapy, leading to increased morbidity and to a substantial economic burden. An appropriate perioperative antibiotic regimen can significantly reduce the incidence of PJIs; however, there is no consensus regarding the optimal antibiotic management for these procedures. Current clinical practice widely varies in terms of the type, duration, and route of antibiotic administration [1].

Recent advances in bioengineering with the introduction of silver-coated implants and the development of novel antibiotics have provided clinicians with additional preventive and treatment measures against PJIs. However, the presence of hematoma, edema, and ischemic tissue may reduce the effectiveness of intravenous antibiotics by impairing diffusion into the affected anatomical area [6]. Since local antibiotic administration allows for higher concentrations at the surgical site with less systemic toxicity compared to systemic administration, it offers an attractive alternative for reducing postoperative infections [7]. Antibiotic-loaded cement is a common strategy for local administration of antibiotics, with mixed results in the literature [8,9,10]. Recent studies indicate that local administration of vancomycin may reduce the incidence of surgical site infections (SSIs) in spinal surgeries, though the efficacy of similar local vancomycin regimens in bone tumor resection surgeries has not been extensively studied [11,12].

Vancomycin, a widely available and inexpensive antibiotic, is a suitable option for local administration in orthopedic surgeries, due to several reasons. First, it is highly active against Gram-positive pathogens, especially against *S. aureus* and Coagulase Negative Staphylococci (CNS), which are the most common culprits for PJIs. Second, vancomycin is stable in powder form, while this is not the case for many other antibiotics. Moreover, local administration of vancomycin can achieve very high concentrations that exceed the minimum inhibitory concentrations (MICs) of Gram-positive bacteria, as opposed to other antibiotics such as gentamycin, for example, which is associated with resistance when subtherapeutic concentrations are evident. Last, vancomycin has a low local tissue toxicity, while other antibiotics such as aminoglycosides may harm the cartilage or interfere with osteoblast activity.

The aim of our study was to evaluate the role of additional local vancomycin administration in reducing the incidence of postoperative periprosthetic infections following bone tumor resection and megaprosthetic reconstruction.

## 2. Results

Initially, 281 consecutive patients who underwent bone tumor resection and megaprosthetic reconstruction in our hospital were considered eligible for this study. However, five patients were excluded due to pre-existing infections at the surgical site, and the final study population consisted of 276 patients. The control group included 142 patients, while the vancomycin group included 134 patients. The two groups were well-balanced in terms of sex (males: 52.1% vs. 49.2%, *p* = 0.63), age (medians: 51.0 vs. 52.5 years, *p* = 0.91), and BMI (medians: 26 vs. 26 kg/m^2^, *p* = 0.13). Demographics of the study cohort are presented in Table 1.

Regarding the type and location of bone tumors, the most common type was osteosarcoma (36.6% in the control group vs. 38.8% in the vancomycin group, *p* = 0.71), while the most common location was the proximal femur (41.5% in the control group vs. 39.5% in the vancomycin group, *p* = 0.80). The second most common type of bone tumor was chondrosarcoma (27.4% in the control group vs. 21.6% in the vancomycin group, *p* = 0.48), while the second most common location was distal femur (19.1% in the control group vs. 29.1% in the vancomycin group, *p* = 0.06). Overall, there was no difference regarding the type of tumor between the two groups. However, the two groups differed in terms of location, since pelvic tumors were most common in the control group compared to the vancomycin group (14.7% vs. 5.2%, *p* = 0.009). The type and location of tumors are presented in Table 2 and Table 3, respectively.

Regarding treatment-related parameters, the length of the resected bone segment was similar between patients who received and those who did not receive local vancomycin (medians: 16 vs. 15 cm, *p* = 0.10), while the median duration of surgery was also similar between the two groups (medians: 160 vs. 150 min, *p* = 0.15). Moreover, soft tissue flaps for wound coverage were used in nine (6.3%) patients in the control group and eleven (8.2%) patients in the vancomycin group (*p* = 0.54). Last, 82 (57.7%) patients in the control group and 65 (48.5%) patients in the vancomycin group received chemotherapy (*p* = 0.12), while radiotherapy was evident in 38 (26.7%) patients in the control group and 29 (21.6%) patients in the vancomycin group (*p* = 0.32). Treatment parameters are shown in Table 4.

### Infection Rates and Isolated Pathogens

At 2 years, twenty-eight patients developed infection in the control group (infection rate: 19.7%, 95% confidence interval [CI]: 13.5–27.2%), while eight patients in the vancomycin group developed infection (infection rate: 5.9%, 95% CI: 2.6–11.4%). The infection rate in the control group was higher than that in the vancomycin group (*p* = 0.001). Regarding the time onset of the infections, there were 15 early infections (<3 months) and 21 delayed infections (3–24 months). Wound healing problems including wound dehiscence or delayed wound healing were present in 13 (9.1%) patients in the control group and in 12 (8.9%) patients in the vancomycin group (*p* = 0.99). The most common isolated pathogens in the 36 patients with infections were coagulase-negative staphylococci (CNS) (n = 19; 52.7%), followed by *Staphylococcus aureus* (n = 8; 22.2%) and *Acinetobacter baumanni* (n = 6; 17.6%). Regarding those infections from coagulase-negative staphylococci, the most common pathogen was methicillin-resistant *Staphylococcus epidermidis* (n = 13, 38.2%), while methicillin-susceptible *Staphylococcus epidermidis* was evident in four cases (11.1%). Among those eight patients with *Staphylococcus aureus* infections, methicillin-resistant *Staphylococcus aureus* was isolated in two (5.5%) patients, while methicillin-susceptible *Staphylococcus aureus* was isolated in two patients (5.5%), and vancomycin-resistant *Staphylococcus aureus* also in two (5.5%) patients. Monomicrobial infections were evident in 19 (52.8%) patients, while polymicrobial infections (>1 pathogen) were evident in 17 patients (47.2%; Table 5).

**Figure 1:**
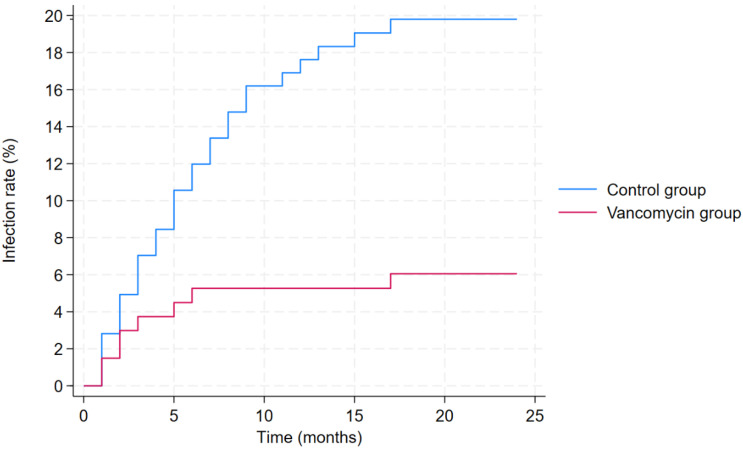
The Kaplan–Meier curves of the two groups, control group that did not receive local vancomycin powder (in blue) and vancomycin group (in red).

The lower infection rate in the vancomycin group was further confirmed by the results of the multivariable competing risks regression analysis, adjusted for sex, age, BMI, type of tumor, location of tumor, and prior chemotherapy (Table 6). Based on the results of this analysis, local vancomycin use was associated with a lower infection risk (hazard ratio [HR]: 0.40, 95% CI: 0.16–0.95, *p* = 0.040; Figure 1). Moreover, the location of the bone tumor also had a significant impact on infection risk, with pelvic tumors being associated with higher infection risk (HR: 5.82, 95% CI: 3.02–11.23, *p* < 0.001).

## 3. Discussion

Pathophysiology of PJI involves development of bacteria in biofilms. As opposed to planktonic bacteria, these biofilms consist of microbial colonies and cells that adhere to a surface or to each other, embedded in a matrix of extracellular substances, and exhibit a distinct phenotype with respect to growth rate and gene expression [13]. Unlike bacteria grown in planktonic cultures, those in biofilms show significantly increased resistance to antibiotics. Therefore, local administration of antibiotics at the time of surgery may be an advantageous strategy that could prevent the formation of biofilms, thus lowering the risk of developing periprosthetic joint infections. Vancomycin is a glycopeptide antibiotic that is widely used against Gram-positive infections. It has been reported that local administration of vancomycin could result in a local concentration of nearly 1000-fold higher than the minimum inhibitory concentration for coagulase-negative staphylococci and methicillin-resistant *Staphylococcus aureus* [14,15]. This is a large retrospective study evaluating the effectiveness of local vancomycin in patients undergoing musculoskeletal tumor resection and megaprosthetic reconstruction. Indeed, based on the results of our study, additional local administration of vancomycin in patients undergoing musculoskeletal tumor resection and megaprosthetic reconstruction was associated with a lower infection risk compared to the control group at 2 years follow-up.

The most commonly isolated bacteria in our study included *Staphylococcus* spp., with the most prevalent coagulase-negative *Staphylococcus* being *S. epidermidis*, followed by *S. lugdunensis*. These pathogens are part of the normal skin flora in the perineal and hip regions, while *S. lugdunensis* frequently mutates, resulting in genetically modified colonies, and its pathogenicity resembles that of *Staphylococcus aureus*. Moreover, polymicrobial infections occurred in about 25% of infections following bone tumor resection surgeries [16,17]. The reported microbiology for PJI in the literature is in line with the pathogens isolated in our cohort, since the most common isolated pathogens in our study were coagulase-negative staphylococci (CNS) (>50% of cases), with *Staphylococcus aureus* being the second most common causative microorganism (23.5%). Moreover, similar to the reported microbiology in tumor surgery, polymicrobial infections were also very common in our study, with 38% of the infected cases having >2 isolated pathogens. Interestingly, among the eight patients who developed infection in the vancomycin group, only two had positive cultures for Gram-positive *Staphylococcus* spp., while in the remaining six patients, Gram-negative pathogens were isolated. This finding further supports the preventive role of vancomycin against the development of PJI from Gram-positive pathogens such as *Staphylococcus* spp. While other antibiotics such as gentamicin or colistin that have a wider spectrum against Gram-negative bacilli could be beneficial to prevent PJIs due to these pathogens, their local administration in orthopedic oncology is less studied, while concerns arise regarding cytotoxicity and their impact on wound healing. Therefore, future research should investigate the potential benefit of combination regimens that include antibiotics against both Gram-positive and Gram-negative pathogens.

In orthopedic oncology, only a few studies have been conducted evaluating the efficacy of local vancomycin in tumor surgeries, reporting mixed results [18,19]. Andreani et al. evaluated 50 patients who underwent bone tumor resection and megaprosthetic reconstruction, comparing the infection rate between 22 patients who received 2 g of vancomycin powder (1 g on the implant surface and 1 g on the tissue layer) and 28 patients who served as the control group [18]. The authors reported that none of the patients in the vancomycin group developed PJIs, while the infection rate in the control group was 21.4%, concluding that local vancomycin may have a preventive role for PJIs. Gutowski et al. also investigated the role of local vancomycin in a series of 54 patients who underwent surgery for musculoskeletal tumors [19]. Similar to the findings of Andreani et al., the authors of this study also reported that local vancomycin may have a beneficial effect against infections, since the infection rate in the vancomycin group was 3.7% as opposed to a 9% infection rate in the control group [18]. However, the authors of both these studies did not conduct any multivariable regression analysis accounting for confounding factors; thus, the revealed differences may be related to various other treatment-related or tumor-related parameters. In another study, Hashimoto et al. described a novel strategy of wrapping the megaprosthetic implants with vancomycin-containing cement in order to prevent postoperative infections in five patients [8]. Although the authors of this study reported that none of the included patients developed PJIs, further studies are needed in order to evaluate whether this strategy is associated with a lower infection rate, since the small number of patients limits the strength of the reported outcomes. In the largest study so far evaluating the efficacy of local vancomycin in orthopedic oncology, Byregowda et al. retrospectively analyzed 221 patients who received only intravenous perioperative antibiotics, and 254 patients who additionally received 1 g of local vancomycin prior to wound closure [20]. The authors of this study compared the infection rate between the two groups and found that there was no difference between the two groups. However, as opposed to our study, megaprosthetic implants were used only in some cases, while most patients in this study underwent reconstruction with allografts or just internal fixation without reconstruction. However, the postoperative infection incidence when megaprosthetic implants are used significantly differs compared to internal fixation or other types of reconstructions. This may be the cause of the lower infection rates that were presented in this study (6% in the control group vs. 8% in the vancomycin group) compared to the reported infection rates in the literature regarding oncologic reconstructions. Another reason for these lower rates may be their definition of infection, which included only cases requiring surgical intervention. The different types of implants that were used in this study, along with the slightly different definition of infection, could be the causes for the different findings compared to our results.

There are concerns regarding the safety of local administration of antibiotics, since there is some evidence indicating that local vancomycin may be associated with adverse events, mainly regarding wound healing problems [21,22]. The low pH of vancomycin solution may lead to skin necrosis, while local inflammatory response may develop as a response to local application of vancomycin. Based on the results of the recent meta-analysis by Xie et al., local vancomycin was associated with a higher incidence of aseptic wound complications (OR: 1.52, 95% CI: 1.04–2.21, *p* = 0.03), while it also prolonged wound healing (OR: 1.93, 95% CI: 1.31–2.85, *p* = 0.001) [22]. In another study comparing the infection rates between patients who received and those who did not receive vancomycin powder following total hip arthroplasty, it was found that wound complications were evident in 4.4% of patients who received local vancomycin [21]. As opposed to these findings, local vancomycin was not associated with a higher rate of wound complications in our study. Safe use of local vancomycin in orthopedic oncology was also supported by the findings of another study including patients with bone tumors, in which vancomycin was used in 22 patients who underwent bone tumor resection, without any reported side effects [18].

There are certain limitations of this study that should be addressed. First, this is a retrospective study including several different types of tumors in various locations; therefore, patients were not randomly assigned; thus, the results may be confounded by several tumor-related or treatment-related parameters. For example, pelvic location was more prevalent in the control group, with a higher rate of infection. However, in order to adjust our evaluated association between vancomycin use and infection development for potential confounding factors, a multivariable regression analysis was performed that was adjusted for sex, age, Body Mass Index (BMI), type of tumor, location of tumor (including pelvic cases), and prior chemotherapy. Moreover, the allocation of patients to either group was based on surgeons’ preference regarding the use of local vancomycin, rather than on randomization. This may introduce an element of selection bias, an inherent limitation of retrospective cohort studies. However, no differences in patient or tumor characteristics were identified between the two groups. Another limitation is that the selected dose of 1 g of local vancomycin in our study may be insufficient to prevent infections in these types of surgeries. Since these surgeries are associated with large dead spaces and long incisions, many surgeons advocate a higher dose of 2 g. However, the efficacy of a higher vancomycin dose was not evaluated in our study; thus, further studies are needed in order to validate whether a higher dose may lead to a further decrease in the infection rate. Moreover, it should be noted that although the 2018 ICM definition for PJI is widely accepted, recent studies suggest that other criteria such as the European Bone and Joint Infection Society (EBJIS) criteria may be more sensitive in detecting low-grade or culture-negative infections. Therefore, our reliance on the ICM definition for PJI may have underestimated the true incidence of PJIs in our cohort. Last, detailed information on certain prosthesis-related parameters that could affect the infection risk, such as cementation status, coatings, and number of prostheses, is missing. It should be noted, though, that none of the implants that were used in this study had antibacterial coatings. Moreover, the number of prostheses is intrinsically related to the anatomical location of surgery, which was taken into consideration in our analysis.

## 4. Methods

A retrospective cohort study was conducted in the largest referral center for musculoskeletal tumors nationwide, including patients who underwent bone tumor resection and megaprosthetic reconstruction of the pelvis and long bones in upper and lower limbs between December 2016 and June 2023. The study protocol was approved by the Institutional Review Board of the hospital (Ref no: 907/20 December 2023), while requirement for a written informed consent was waived due to the retrospective nature of the study. Patients with pre-existing infections at the surgical site were excluded. Patients were divided into two groups: Group A consisted of patients receiving only perioperative antibiotic treatment according to the hospital protocol, while Group B included patients receiving the same perioperative antibiotic regime along with additional local vancomycin powder applied to the surgical area during wound closure. Patients were allocated to the control or vancomycin group based on the preference of the operating surgeon regarding the use of local vancomycin. Therefore, patients who were operated on by surgeons who routinely applied local vancomycin powder were assigned to the vancomycin group, while patients who were operated on by surgeons who did not use local vancomycin were assigned to the control group. Group allocation was therefore based on practice patterns rather than patient- or tumor-specific factors. Patient data were recorded in a database including demographics (age, sex, BMI, smoking status, etc.), tumor-related parameters (histological type, anatomical location, etc.), treatment-related parameters (resection length, adjuvant chemotherapy, radiotherapy, other surgical details), and parameters related to infection (pathogen, time of infection onset, etc.). The primary event of interest was the development of periprosthetic infections at 2 years’ follow-up.

The perioperative antibiotic protocol included 400 mg of IV teicoplanin 30 min before incision. Postoperatively, 200 mg of teicoplanin once daily and 4 (+1.5) g of piperacillin/tazobactam every 8 h were administered intravenously until the fifth postoperative day. In shoulder surgery, patients received teicoplanin and clindamycin for 5 days. Patients in Group B received an additional 1 g of vancomycin powder locally in the soft tissue of the wound during layered closure. Based on our protocol, commercially available sterile vancomycin powder (1 g) was used directly from its vial without dilution. The powder was evenly sprinkled over the soft tissues around the prosthesis and within the wound bed during layered closure. The surgical drain was clamped for 60 min post-closure to maximize local exposure and prevent aspiration of vancomycin powder.

Diagnosis of infection was based on the 2018 International Consensus Meeting (ICM) criteria for periprosthetic joint infections [23]. According to these criteria, an infection is defined based on several parameters such as culture results, synovial fluid findings including synovial fluid white blood cell count, and serological biomarkers such as C reactive protein (CRP), erythrocyte sedimentation rate (ESR), and D-dimer levels. Specifically, periprosthetic joint infection is defined when at least one of two major criteria is present (two positive periprosthetic cultures with phenotypically identical organisms or a sinus tract communicating with the joint) or when a combination of minor criteria is present (including elevated CRP/ESR, elevated synovial leukocyte count or neutrophil percentage, positive histology, or a single positive culture). The 2018 ICM criteria are in line with the updated CDC criteria for organ/space surgical site infections [24]. These criteria were selected because they represent the most widely adopted and internationally validated criteria for PJI diagnosis. This also ensures comparability with prior literature and consensus-based reproducibility.

Regarding pathogen identification, all tissue and fluid samples that were obtained postoperatively were cultured on standard aerobic and anaerobic media such as blood agar, chocolate agar, and MacConkey agar. Moreover, Sabouraud dextrose agar was used for fungal isolation, while Löwenstein–Jensen medium and Mycobacteria Growth Indicator Tubes were used for mycobacterial isolation. All cultures were incubated at 35–37 °C for up to 14 days to allow growth of slow-growing and fastidious organisms, except for those involved in mycobacterial isolation which were incubated for up to 6 weeks. Preliminary identification of pathogens was performed using conventional biochemical tests including catalase, coagulase, and oxidase assays. Final species-level identification and antimicrobial susceptibility testing were carried out using the VITEK2 automated system. All results were interpreted based on the European Committee on Antimicrobial Susceptibility Testing (EUCAST) guidelines.

### Statistical Analysis

Descriptive statistics were used to summarize patient demographics, tumor-related characteristics, microbiological data, and treatment parameters. Continuous variables were presented as medians with interquartile ranges (IQRs) for non-normally distributed data or means with standard deviations (SDs) for normally distributed variables; categorical variables were reported as counts and percentages. Group comparisons were performed using the Wilcoxon rank-sum (Mann–Whitney) test for continuous variables and the chi-square test (or Fisher’s exact test for expected cell counts < 5) for categorical variables. Infection rates were compared between groups using the chi-square test. Time-to-event analysis was conducted using the Kaplan–Meier method to estimate cumulative infection incidence. To account for death as a competing risk, a competing risks regression analysis (Fine and Gray method) was employed; patients were censored at loss to follow-up. A multivariable model adjusted for sex, age, BMI, tumor type, tumor location, and prior chemotherapy (variables selected a priori based on clinical relevance) was used. The proportional hazards assumption was tested using Schoenfeld residuals and upheld. All analyses were performed using Stata 19 (StataCorp, College Station, TX, USA), with a two-sided *p*-value < 0.05 considered statistically significant.

## 5. Conclusions

Megaprosthetic reconstructions following bone tumor resections are associated with a high risk of PJI due to several reasons, such as prolonged duration of surgery and compromised immunity systems of patients. Therefore, several preventive measures have been evaluated, with local administration of antibiotics being a promising strategy. Vancomycin is a glycopeptide antibiotic that has proven efficacy towards Gram-positive bacteria; thus, local administration may be effective against pathogens causing PJIs. Indeed, our results suggest that local use of vancomycin powder in the soft tissue of the wound during layered closure may be associated with a lower infection risk in patients undergoing megaprosthetic reconstruction following bone tumor resection. However, larger high-quality studies with a randomization methodology are needed to evaluate whether this strategy is advantageous in preventing infections, following these extensive oncologic surgeries.

## Figures and Tables

**Table 1:** Demographics of the study population.

Variables	Control Group (n = 142)	Vancomycin Group (n = 134)	*p*-Value
Age (years)	51 (24–66)	52.5 (47–55)	0.91
Sex (males, %)	74 (52.1)	66 (49.2)	0.63
Smoking status *	13 (9.1)	15 (11.1)	0.57
Body Mass Index (kg/m^2^)	26 (24–27)	26 (25–29)	0.13

* Current active smoking at the time of surgery, irrespective of pack-years. Data are reported as medians (interquartile ranges) or as counts (percentages) when appropriate.

**Table 2:** Type of tumors.

Tumor	Control Group (n = 142)	Vancomycin Group (n = 134)	*p*-Value
Osteosarcoma	52 (36.6)	52 (38.8)	0.71
Chondrosarcoma	39 (27.4)	29 (21.6)	0.48
Ewing	13 (9.1)	11 (8.2)	0.83
Giant-cell tumor	2 (1.4)	5 (3.7)	0.27
Other	7 (4.9)	6 (4.4)	0.99
Metastatic tumor	29 (20.4)	31 (23.1)	0.66

Data are reported as counts (percentage).

**Table 3:** Location of tumors.

Location	Control Group (n = 142)	Vancomycin Group (n = 134)	*p*-Value
Proximal femur	59 (41.5)	53 (39.5)	0.80
Distal femur	27 (19.1)	39 (29.1)	0.06
Knee	2 (1.4)	4 (2.9)	0.43
Proximal tibia	10 (7.0)	6 (4.4)	0.44
Proximal humerus	23 (16.2)	25 (18.6)	0.63
Pelvis	21 (14.7)	7 (5.2)	0.009

Data are reported as counts (percentage).

**Table 4:** Treatment-related parameters.

Variables	Control Group (n = 142)	Vancomycin Group (n = 134)	*p*-Value
Preoperative radiotherapy	38 (26.7)	29 (21.6)	0.32
Preoperative chemotherapy	82 (57.7)	65 (48.5)	0.12
Duration of surgery (min)	150 (130–190)	160 (140–190)	0.15
Use of soft tissue flaps	9 (6.3)	11 (8.2)	0.54
Length of resection (cm)	15 (13–18)	16 (13.5–18)	0.10

Data are reported as medians (interquartile ranges) or as counts (percentages) when appropriate.

**Table 5:** Isolated pathogens in periprosthetic infections.

Pathogens	Patients (n = 36)	Vancomycin Group (n = 8)	Control Group (n = 28)
Methicillin-resistant *Staphylococcus epidermidis*	7 (19.4%)	0 (0.0%)	7 (25.0%)
Methicillin-sensitive *Staphylococcus epidermidis*	3 (8.3%)	0 (0.0%)	3 (10.7%)
*Staphylococcus lugdunensis*	1 (2.8%)	0 (0.0%)	1 (3.6%)
Methicillin-resistant *Staphylococcus aureus*	2 (5.5%)	0 (0.0%)	2 (7.1%)
Vancomycin-resistant *Staphylococcus aureus*	2 (5.5%)	2 (25.0%)	0 (0.0%)
Methicillin-sensitive *Staphylococcus aureus*	1 (2.8%)	0 (0.0%)	1 (3.6%)
*Acinetobacter baumannii*	2 (5.5%)	2 (25.0%)	0 (0.0%)
*Enterobacter clocae*	1 (2.8%)	0 (0.0%)	1 (3.6%)
Methicillin-resistant *Staphylococcus epidermidis* + Methicillin-sensitive *Staphylococcus aureus* + *Pseudomonas aeruginosa* + *Enterobacter clocae* + *Methicillin-resistant Staphylococcus aureus* + *Candida* spp., *Pseudomonas aeruginosa* + *Candida* spp., methicillin-resistant *Staphylococcus aureus*	1 (2.8%) 1 (2.8%) 1 (2.8%) 1 (2.8%) 1 (2.8%) 1 (2.8%)	0 (0.0%) 0 (0.0%) 0 (0.0%) 0 (0.0%) 0 (0.0%) 0 (0.0%)	1 (3.6%) 1 (3.6%) 1 (3.6%) 1 (3.6%) 1 (3.6%) 1 (3.6%)
Methicillin-sensitive *Staphylococcus epidermidis* + *Candida* spp.	1 (2.8%)	0 (0.0%)	1 (3.6%)
*Staphylococcus capitis* + *Aspergillus* spp.	1 (2.8%)	0 (0.0%)	1 (3.6%)
*Acinetobacter baumannii* + *Enterococcus faecium* + *Proteus mirabilis* + *Klebsiella pneumoniae*	1 (2.8%) 2 (5.6%) 1 (2.8%)	0 (0.0%) 1 (12.5%) 1 (12.5%)	1 (3.6%) 1 (3.6%) 0 (0.0%)
*Streptococcus oralis*	1 (2.8%)	0 (0.0%)	1 (3.6%)
*Enterobacter clocae* + *Proteus mirabilis*	2 (5.6%)	1 (12.5%)	1 (3.6%)
*Enterococcus faecium* + *Klebsiella pneumoniae* + *Candida* spp.	1 (2.8%)	0 (0.50%)	1 (3.6%)
*Achromobacter* spp. + *Pseudomonas aeruginosa*	1 (2.8%)	1 (12.5%)	0 (0.0%)

Data are presented with frequency (percentage).

**Table 6:** Results of competing risk regression (dependent variable: infection occurrence) with age, sex, BMI, type of tumor, tumor location (pelvis vs. other locations), vancomycin use, and prior chemotherapy as independent variables.

	Infection		
	HR	(95% CI)	*p*-Value
Age	0.98	(0.97–1.004)	0.058
Sex	0.93	(0.48–1.77)	0.82
Body Mass Index	0.99	(0.91–1.09)	0.97
Type of tumor	0.93	(0.79–1.12)	0.46
Location (pelvis)	5.82	(3.02–11.2)	<0.001
Vancomycin use	0.40	(0.16–0.95)	0.040
Prior chemotherapy	1.25	(0.63–2.49)	0.51

Abbreviations: HR, hazard ratio; CI, confidence interval.

## Data Availability

The raw data supporting the conclusions of this article will be made available by the authors on request.
